# P53 suppresses SENP3 phosphorylation to mediate G2 checkpoint

**DOI:** 10.1038/s41421-020-0154-2

**Published:** 2020-04-21

**Authors:** Yang Wang, Jing Tian, Chao Huang, Jiao Ma, Gaolei Hu, Yalan Chen, Tianshi Wang, Rong Cai, Yong Zuo, Hongsheng Tan, Qiuju Fan, Baijun Dong, Wei Xue, Jing Yi, Guoqiang Chen, Jun Tu, Jinke Cheng

**Affiliations:** 1 Department of Biochemistry and Molecular Cell Biology, Shanghai Key Laboratory for Tumor Microenvironment and Inflammation, Shanghai Jiao Tong University School of Medicine, 200025 Shanghai, China; 2 Key Laboratory of Cell Differentiation and Apoptosis of Chinese Ministry of Education, Shanghai Jiao Tong University School of Medicine, 200025 Shanghai, China; 3 Department of Urology, Renji Hospital affiliated Shanghai Jiao Tong University School of Medicine, 200025 Shanghai, China; 4 Thoracic Oncology Institute at Shanghai Chest Hospital, Shanghai Jiao Tong University, Shanghai, China; 5 School of Pharmacy, Shanghai University of Traditional Chinese Medicine, 201203 Shanghai, China

**Keywords:** Sumoylation, Cell division

## Abstract

In response to DNA damage, p53-mediated signaling is regulated by protein phosphorylation and ubiquitination to precisely control G2 checkpoint. Here we demonstrated that protein SUMOylation also engaged in regulation of p53-mediated G2 checkpoint. We found that G2 DNA damage suppressed SENP3 phosphorylation at G2/M phases in p53-dependent manner. We further found that the suppression of SENP3 phosphorylation was crucial for efficient DNA damage/p53-induced G2 checkpoint and G2 arrest. Mechanistically, we identified Cdh1, a subunit of APC/C complex, was a SUMOylated protein at G2/M phase. SENP3 could de-SUMOylate Cdh1. DNA damage/p53-induced suppression of SENP3 phosphorylation activated SENP3 de-SUMOylation of Cdh. De-SUMOylation promoted Cdh1 de-phosphorylation by phosphatase Cdc14B, and then activated APC/C^Cdh1^ E3 ligase activity to ubiquitate and degrade Polo-like kinase 1 (Plk1) in process of G2 checkpoint. These data reveal that p53-mediated inhibition of SENP3 phosphorylation regulates the activation of Cdc14b-APC/C^Cdh1^-Plk1 axis to control DNA damage-induced G2 checkpoint.

## Introduction

Cells have developed a set of surveillance machinery to ensure precise DNA replication, chromosome segregation, and allocation to daughter cells^1^. Among them, G2 DNA damage checkpoint is essential for precise cell division and cell survival. Under genotoxic stresses, cells initiate DNA damage response (DDR), which arrests cells at the G2 phase and delays entry into mitosis until DNA lesions are corrected^2^. Although cells with mutant p53 can be temporally arrested at the G2 phase, however, wild-type p53 is required for efficient and prolonged arrest at the G2 phase in response to DNA damage^2^. One of the mechanisms by which p53 arrests cells at the G2 is by inhibiting Cyclin-dependent kinase 1(Cdk1)^3^, the activity of which is simultaneously suppressed by Gadd45, p21, and 14–3–3σ, all of which are transcription targets of p53^4–6^. Other pathways that inhibit Cdk1 include ATR-Chk1 signaling. Upon DNA damage, ATR-Chk1 cascade is activated, and then sequesters Cdc25A in the cytoplasm where it cannot de-phosphorylate and activate Cdk1/Cyclin B^7,8^, or degrade Cdc25A via the SCF^βTrcp ^^9–12^, which is fundamentally crucial for cancer development^13^. Thus, Chk1 activation results in the attenuation of Cdk1 activity by increasing inhibitory phosphorylation to cause G2 arrest in response to DNA damage.

During cell cycle progression, the ubiquitin ligase anaphase-promoting complex or cyclosome (APC/C^Cdh1^) usually remains inactive at the G2 phase, and becomes active at late mitosis and G1 phase to prevent premature progression into S phase^14,15^. Recently, it has been reported that under the genotoxic stress, G2/M cell cycle transition regulatory network members Cdc14, APC/C^Cdh1^, and Plk1 are engaged in the DNA damage-induced G2 cell cycle arrest^16–18^. During this process, phosphatase Cdc14B translocates from the nucleolus to nucleoplasm leading to dephosphorylation and activation of APC/C^Cdh1^, which then target Plk1 for degradation^16^. This process further induces the stabilization of Wee1, which phosphorylates Cdk1 at Tyr15 for inactivation^19^, and stabilization of Claspin to mediate activation of Chk1^20,21^, both of which eventually lead to efficient G2 arrest.

Protein SUMOylation emerges as an important regulatory mechanism in a variety of cellular functions such as DNA replication, gene expression, and chromosome condensation and segregation to ensure smooth progression of cell cycle^22,23^. De-SUMOylation protease SENP regulates these processes by de-SUMOylation^24–26^. For example, UV and H_2_O_2_ promote SENP1 association and de-SUMOylation of Sirt1 to enhance p53-dependent apoptosis^27^. SENP2 has a crucial role in the control of hnRNP-K function as a p53 co-activator in response to DNA damage^28^. SENP5 is required for effective ATR activation in HepG2 cells under IR irradiation condition^29^. SENP6-knockdown cells reveal the significant phenotype of DDR and defective cell cycle progression in both S and G2/M phases^30^. SENP7-mediated removal of SUMO2/3 chains from Kap1 is particularly important in the response to DNA damage^31^. We have previously shown that SENP3 is phosphorylated at the G2/M phase, which is required for accurate transition of mitotic cycle^32^. Here we showed that p53 mediated the inhibition of SENP3 phosphorylation in response to genotoxic stress at the G2 phase. Furthermore, we found that the inhibition of SENP3 phosphorylation was essential for Cdh1 de-SUMOylation, which turned on Cdc14B-Cdh1-Plk1 axis leading to G2 checkpoint. Thus, we reveal that the p53-mediated the suppression of SENP3 phosphorylation controls G2 checkpoint in responding to DNA damage.

## Results

### P53 suppresses SENP3 phosphorylation responding to DNA damage

**Fig. 1:**
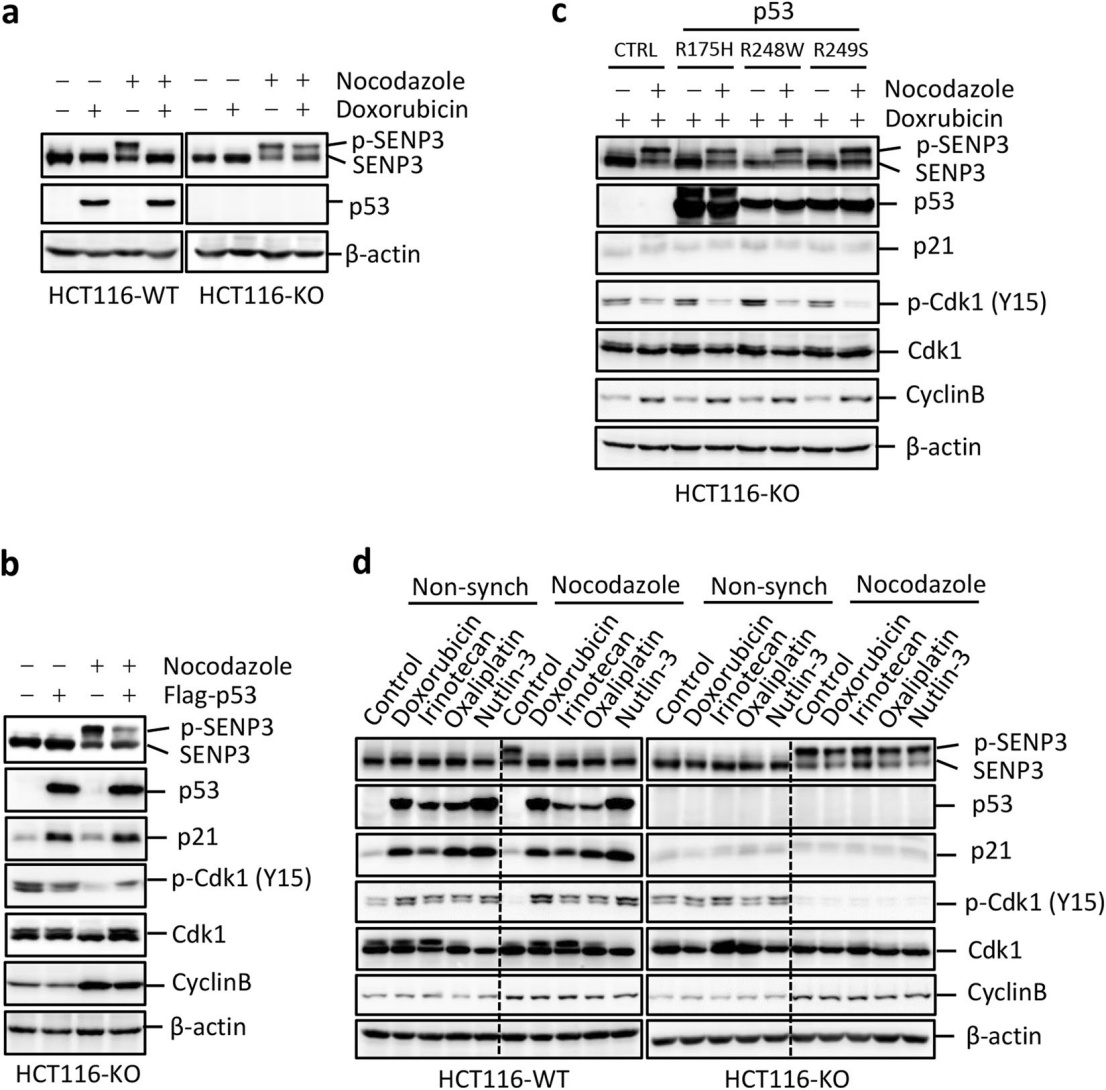
Suppression of SENP3 phosphorylation at G2/M phase by p53 upon DNA damage. **a** HCT116 p53 WT/KO Cells were synchronized or not at G1/S by double thymidine block, followed by releasing into fresh or nocodazole-containing medium for 7 h. Cells were then pulsed with either solvent or doxorubicin for 1 h and cultured for additional 3 h before harvest. Whole-cell extracts were analyzed by western blot with antibodies to the indicated proteins. **b** HCT116 p53 KO cells were transfected with Flag-p53 plasmids, and then cells were treated as in Fig. 1a. Whole-cell extracts were analyzed by western blot with antibodies to the indicated proteins. **c** Various p53 mutant plasmids were transfected into HCT116 KO cells, and then cells were treated as in Fig. 1a. Whole-cell extracts were analyzed by western blot with antibodies to the indicated proteins. **d** HCT116 p53 WT / KO cells were synchronized or not as in **a**, and then cells were treated with the indicated chemotherapy drugs. Whole-cell extracts were analyzed by western blot with antibodies to the indicated proteins.

We have shown that SENP3 but not SENP5 (Supplementary Fig. S1a) is phosphorylated by Cdk1 to inactivate its de-SUMOylation activity at the G2/M phase of the cell cycle^32^. However, it was unknown how to regulate SENP3 phosphorylation via Cdk1. P53 as a key cell cycle inhibitor can suppress Cdk1 activity in response to DNA damage^2,3^, we thus reasoned that p53 would be an upstream regulator to modulate SENP3 phosphorylation responding to DNA damage. To this end, we carried out an assay in p53 wild-type HCT116 (HCT116 WT) and p53 deficiency HCT116 (HCT116 KO) cell lines, Both cells were synchronized at the G1/S phase by thymidine block and then released into fresh or nocodazole-containing media for 7 h to reach G2 phase, followed by doxorubicin treatment for 1 h and culture for additional 3 h before harvest (defined as G2 DDR protocol, which was used in the most of following experiments). As shown in Fig. 1a, nocodazole treatment induced SENP3 phosphorylation in both cells. Addition of doxorubicin-induced p53 expression in HCT116 WT but not in HCT116 KO at G2 phase. Importantly, we found that doxorubicin treatment suppressed SENP3 phosphorylation in HCT116 WT cells but not in HCT116 KO cells. We confirmed it by showing that silencing p53 in HCT116 WT would rescue doxorubicin-induced suppression of SENP3 phosphorylation at G2 phase (Supplementary Fig. S1b). Furthermore, over-expression of p53 in HCT116 KO cells, which recovered the induction of p21 expression and down-regulated Cdk1 activity indicated by the increased inhibitory phosphorylation in Cdk1 at Tyr15, could suppress SENP3 phosphorylation at G2 phase like HCT116 WT cells (Fig. 1b).

We further tested whether p53 transcriptional activity would be essential for its suppression of SENP3 phosphorylation in G2 DDR. HCT116 KO cells transfected with various p53 mutants were treated with doxorubicin and nocodazole for G2 DDR. As shown in Fig. 1c, p53 transactivation mutants, which could not induce p21 expression and suppress Cdk1 activity, certainly did not inhibit SENP3 phosphorylation in response to genotoxic stress (Fig. 1c). Moreover, we also tested the effect of several chemotherapy drugs, which cause DNA damage, on SENP3 phosphorylation. We found that all these tested drugs could suppress SENP3 phosphorylation in HCT116 WT but not in HCT116 KO cells at G2 phase (Fig. 1d). These data suggest that DNA damage-induced p53 activation suppresses SENP3 phosphorylation at G2 phase.

### P53 inhibition of SENP3 phosphorylation mediates G2 checkpoint

**Fig. 2:**
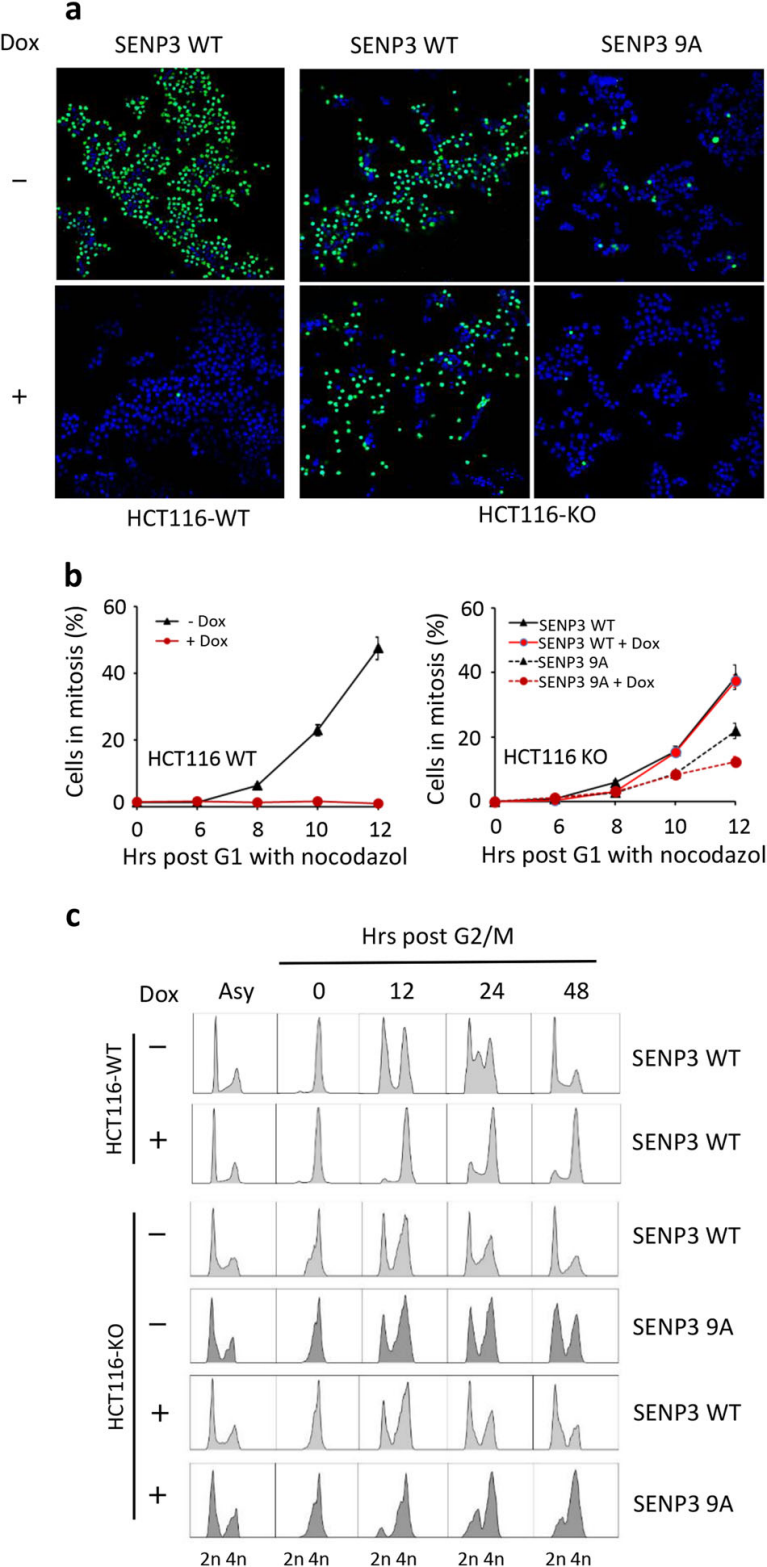
Inhibition of SENP3 phosphorylation by p53 mediates DNA damage-induced G2 arrest. **a** HCT116 p53 WT/KO cells infected with retroviruses either encoding wild-type SENP3 or SENP3 9A were synchronized and treated with or without doxorubicin as in Fig. 1a. Cells were then released and incubated in nocodazole-containing medium to trap cells in mitosis. Cells were analyzed by immunofluorescence with anti- p-H3S10 (green) at 12 h post release. **b** Statistics of results in **a**. Samples were collected at the indicated times, and the percentage of mitotic cells was monitored by immunodetection of Histone H3 phosphorylated on Ser10 using flow cytometry (*n* = 3, ±SD), **P < 0.01. **c** HCT116 p53 WT/KO cells treated as in **a** were released into fresh medium for indicated time and cells were collected for cell-cycle analysis by FACS.

DNA damage induces p53 expression to mediate G2 arrest^2^. We then asked whether p53-mediated inhibition of SENP3 phosphorylation would contribute to p53-mediated G2 arrest. HCT116 KO cells were stably transfected by either SENP3 wild-type (SENP3 WT) or the mutant of SENP3 9 phosphorylation sites (SENP3 9A)^32^. These cells were synchronized at G2 phase, and then treated with or without doxorubicin for 1 h followed by culture in fresh media for 24 h before addition of nocodazole, which traps cells in mitosis. The cells in mitosis were indicated by immunostaining with the phosphorylated Histone H3 at Ser10 (p-H3S10). As shown in Fig. 2a, b, the most of HCT116 WT cells were in mitosis in the condition of without doxorubicin treatment. Upon doxorubicin treatment, almost all HCT116 WT cells were blocked to enter into mitosis (Fig. 2a, b). SENP3 WT expression in HCT 116 KO could not recover doxorubicin-induced G2 arrest because of p53 deficiency. However, SENP3 9A expression blocked HCT116 KO cells entering into mitosis even in the condition of with or without doxorubicin treatment (Fig. 2a, b), indicating that deficiency of SENP3 phosphorylation could mimic p53-mediated G2 arrest.

We further performed flow cytometry analysis to determine whether the inhibition of SENP3 phosphorylation affects cell cycle. We first found that the most of G2-synchronized HCT116 WT cells would progressively go to the pattern that G1 phase cells were the dominant at 48 h after releasing (Fig. 2c). However, doxorubicin treatment caused the most of G2-synchronized HCT116 WT cells still maintained in G2 phase for 48 h (Fig. 2c). Compared to DNA damage/p53-mediated G2 arrest in HCT116 WT cells, HCT116 KO cells transfected with SENP3 WT showed a similar cell cycle pattern as HCT116 WT cells did after releasing from G2-synchronized status in normal condition (Fig. 2c). SENP3 WT-HCT116 KO cells could not recover its G2 arrest in the condition of doxorubicin treatment (Fig. 2c). However, SENP3 9A expression in HCT116 KO cells could recover G2 arrest in the conditions of with or without doxorubicin treatment (Fig. 2c). These data, which were consistent with the results from analysis on mitotic cell staining shown above, suggest that p53-mediated the inhibition of SENP3 phosphorylation engages in DNA damage/p53-induced G2 checkpoint. We also noticed that doxorubicin-treated SENP3 9A-HCT116 KO cells had much more cells arrested at G2 phase than the cells without doxorubicin treatment (Fig. 2c). The reason for this observation might be that DNA damage-caused G2 arrest would have mechanisms other than p53-SENP3-mediated effects.

### Inhibiting SENP3 phosphorylation promotes Plk1-linked signaling in G2 checkpoint

**Fig. 3:**
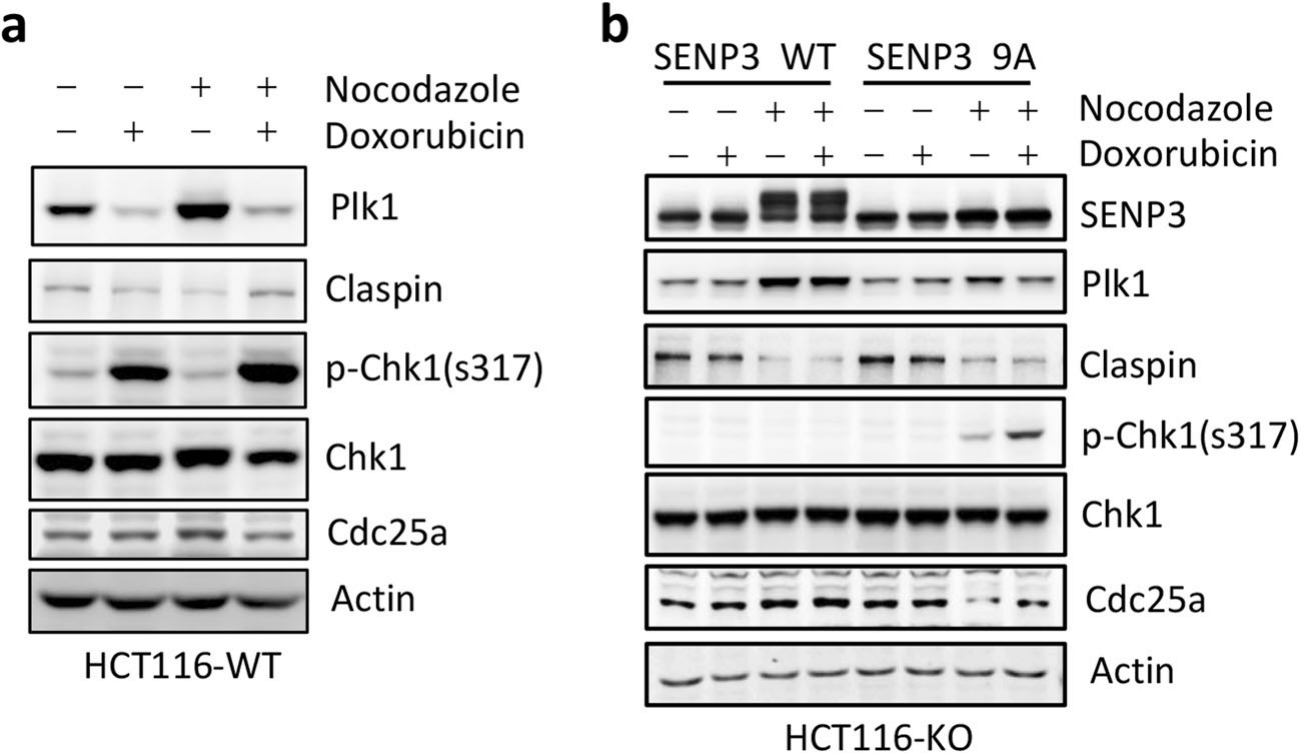
Plk1-Claspin-Chk1 signaling pathway is regulated by phosphorylation of SENP3 at G2. **a** HCT116 p53 WT cells were treated as in Fig. 1a. Whole-cell extracts were analyzed by western blot with antibodies to the indicated proteins. **b** HCT116 p53 KO cells infected with retroviruses either encoding wild-type SENP3 or SENP3 9A were treated as in Fig. 1a. Whole-cell extracts were analyzed by western blot with antibodies to the indicated proteins.

Bassermann et al. reported that in response to DNA damage in G2, activating ubiquitin ligase APC/C^Cdh1^ targets Plk1, a mitotic kinase, for degradation, which reduces the phosphorylation of Claspin, and then induces the stabilization of Claspin leading to Chk1 activation. This Plk1-Chk1 axis would attenuate Cdk1 activity and arrest cells in G2^3,16^. As p53-mediated inhibition of SENP3 phosphorylation contributes DNA damage-induced G2 checkpoint, we hypothesized that SENP3 phosphorylation would modulates Plk1-Chk1 axis in the process of G2 arrest. To this end, we analyzed the alteration of Plk1-Chk1 axis in SENP3 WT- or SENP3 9A-HCT116 KO cells in G2 checkpoint. Doxorubicin treatment could reduce Plk1, increase Claspin and Chk1 phosphorylation, and reduce Cdc25a in G2-synchronized HCT116 WT cells (Fig. 3a). Compared to observations in HCT116 WT, expression of SENP3 WT in HCT116 KO showed the changes as similar as HCT116 WT cells did at G2 phase. Because of p53 deficiency, doxorubicin treatment could not affect SENP3 phosphorylation as well as Plk1/Claspin/Chk1/Cdc25a in G2-synchronized SENP3 WT-HCT116 KO cells (Fig. 3b). Importantly, the expression of SENP3 9A in HCT116 KO cells could reduce Plk1, increase Claspin and the phosphorylated Chk1, and reduce Cdc25a in G2-synchronized HCT116 KO cells (Fig. 3b), which were as similar as the alterations shown in HCT116 WT with doxorubicin treatment. These data indicate that p53-mediated inhibition of SENP3 phosphorylation regulates Plk1-Chk1 signaling in G2 checkpoint.

### SENP3-mediated de-SUMOylation of Cdh1 potentiates activation of APC/C^Cdh1^

**Fig. 4:**
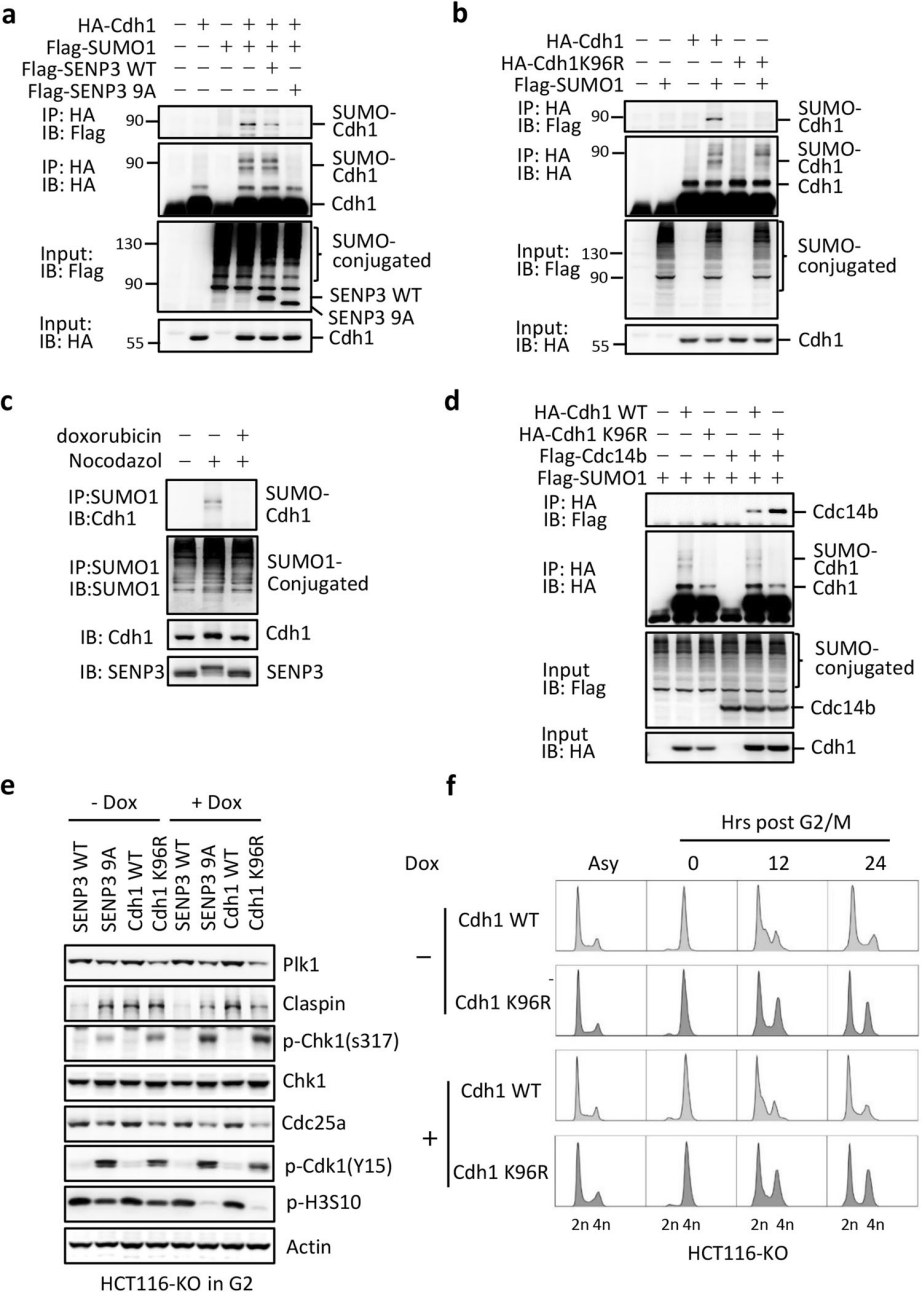
SENP3-mediated de-SUMOylation activates APC/C^Cdh1^. **a** 293T cells were transfected with the indicated plasmids. After 36 h cells were treated with nocodazole for further 12 h. Cells were lysed and immunoprecipitation was performed with HA antibody and western blot was performed with Flag antibody. **b** 293T cells were transfected with the indicated plasmids and were treated as in **a**. Cells were lysed and immunoprecipitation was performed with HA antibody and western blot was performed with Flag antibody. **c** HCT116 p53 WT cells were treated as in Fig. 1a. Cells were lysed and immunoprecipitation was performed with SUMO1 antibody and western blot was performed with indicated antibodies. **d** 293T cells were transfected with the indicated plasmids and were treated as in **a**. Cells were lysed and immunoprecipitation was performed with HA antibody and western blot was performed with Flag antibody. **e** HCT116 p53 KO cells infected with retroviruses encoding either wild-type SENP3 or SENP3 9A, or wild-type Cdh1, or Cdh1 K96R were treated as in Fig. 1a. Whole-cell extracts were analyzed by western blot with antibodies to the indicated proteins. **f** HCT116 p53 KO cells infected with retroviruses encoding either wild-type Cdh1 or Cdh1 K96R were treated as in Fig. 2c. Cells were collected at indicated time points after release and the cell-cycle distribution was measured by FACS.

We previously showed that the phosphorylation would suppress SENP3 de-SUMOylation activity. Therefore, p53-mediated inhibition of SENP3 phosphorylation would activate SENP3 for de- SUMOylation activity. Above data showed that SENP3 9A could reduce Plk1 level in G2-synchronized HCT116 KO cells, suggesting that SENP3 might target the ubiquitin ligase APC/C^Cdh1^ through de-SUMOylation to promote Plk1 ubiquitination for degradation. CDK1 phosphorylates Cdh1 and inhibits its E3 ligase activity. Upon DNA damage at G2, phosphatase Cdc14B would de-phosphate Cdh1, then activate APC/C^Cdh1^ leading to G2 arrest. We thus hypothesized that Cdh1 is a SUMOylated protein and SENP3 could de-SUMOylate Cdh1 to activate APC/C^Cdh1^ in G2 checkpoint. To test it, we did Cdh1 SUMOylation assay in cells transfected with Cdh1 and/or SUMO1. Cdh1 could be conjugated by SUMO1 but not SUMO2 (Supplementary Fig. S2a, b). SENP3 was a de-SUMOylation protease of Cdh1 (Fig. 4a). We detected that SENP3 9A mutant had more activity in de-conjugation of SUMO-Cdh1 proteins than SENP3 WT did (Fig. 4a). We further mapped Lys 96 on Cdh1 as a SUMOylation site. Mutation of Cdh1 K96 to Arg or E98 to Ala completely abolished Cdh1 SUMOylation, indicating K96 as a unique SUMOylation site on Cdh1 (Fig. 4b and Supplementary Fig. S2c).

Furthermore, we tested the responses of several SUMOylated proteins including Cdh1, APC4, and APC7 to nocodazole and/or doxorubicin treatment. We found that SUMOylated-Cdh1 only occurred in G2-synchronized cells (Fig. 4c). The doxorubicin treatment could markedly reduce Cdh1 SUMOylation in these cells. In contrast, both APC4 and APC7 were constitutively modified by SUMO1, and did not response to nocodazole and/or doxorubicin treatment (Supplementary Fig. S3a). Interestingly, we simultaneously detected the de-phosphorylation of both SENP3 and Cdh1 in these doxorubicin-treated G2 HCT116 WT cells (Fig. 4c). Based on these observations, we reasoned that the p53-mediated inhibition of SENP3 phosphorylation enhances its de-SUMOylation of Cdh1 leading to de-phosphorylation of Cdh1 in cells at G2 phase. If this is real, SENP3-mediated de-SUMOylation of Cdh1 should promote Cdc14b recruitment to de-phosphorylate Cdh1. To test it, we compared the binding efficiency of Cdh1 WT or Cdh1 K96R or Cdh1 E98A mutant with Cdc14b. As shown in Fig. 4d and Supplementary Fig. S3b, Cdh1 K96R or E98A mutant could bind with more Cdc14b than Cdh1 WT did (Fig. 4d and Supplementary Fig. S3b). Furthermore, SENP3 9A enhanced the binding of Cdh1 with Cdc14B compared to SENP3 WT in cells at G2 phase (Supplementary Fig. S3c).

To determine the role of SENP3-mediated de-SUMOylation of Cdh1 in the Plk1-engaged signaling, we analyzed the alteration of this signaling in SENP3 9A or Cdh1 K96R-transfected HCT116 KO cells. As shown in Fig. 4e, the expression of SENP3 9A in G2 HCT116 KO cells could induce the Plk1-Chk1 signaling, including reducing Plk1, activating Chk1, reducing Cdc25A, and finally inhibiting CDK1 activity (Fig. 4e). Interestingly, Cdh1 K96R but not Cdh1 WT showed as similar as SENP3 9A did in G2 HCT116 KO cells, suggesting that SENP3-mediated de-SUMOylation of Cdh1 is a key event for APC/C^Cdh1^ activation in controlling Plk1-engaged signaling (Fig. 4e). We also tested Plk1-engaged signaling response to Cdh1 E98A mutant and found that Cdh1 E98A showed the same response to doxorubicin as Cdh1 K96R did (Supplementary Fig. S4). To further determine the role of SENP3-mediated de-SUMOylation of Cdh1 in G2 checkpoint, we analyzed the cell cycle in G2-synchronized HCT116 KO cells transfected with Cdh1 WT or K96R. Cells transfected with Cdh1 K96R had much more cells at G2/M phase after releasing from G2/M, suggesting that SENP3-mediated Cdh1 de-SUMOylation would promote G2 arrest (Fig. 4f). Altogether, these data indicate that SENP3-mediated de-SUMOylation of Cdh1 allows APC/C^Cdh1^ to activate Plk1-Chk1 signaling promoting G2 arrest.

## Discussion

**Fig. 5:**
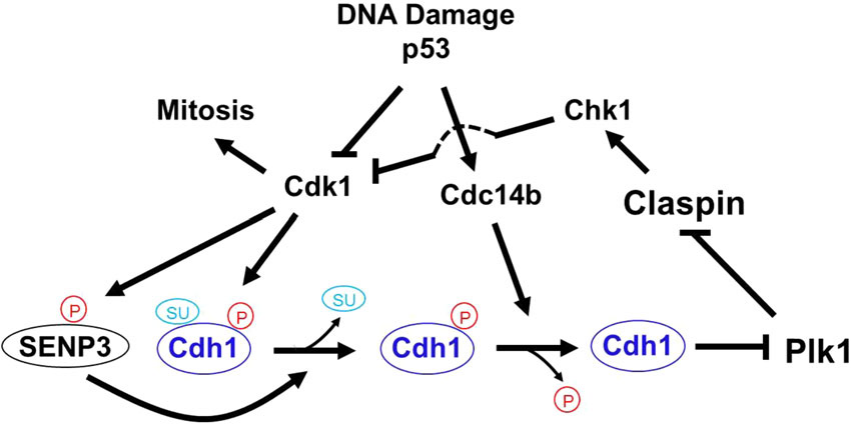
Illustration of the role of SENP3 phosphorylation in DNA damage-induced G2 arrest.

In this study, we explore how phosphorylation regulates SENP3 function in response to DNA damage-induced G2 checkpoint. We find that G2 DNA damage inhibits SENP3 phosphorylation through p53-depend manner. The inhibition of SENP3 phosphorylation promotes its de-SUMOylation of Cdh1 to activate Plk1-Chk1 signaling leading to G2 arrest. Mechanically, the de-SUMOylation of Cdh1 can promote the binding of Cdh1 with phosphatase Cdc14b to de-phosphorylate Cdh1, then activates APC/C^Cdh1^ ubiquitination of Plk1 for degradation, which activates Chk1 leading to Cdc25a degradation and then inhibits Cdk1 activation for G2 checkpoint (Fig. 5). These data reveal that the inhibition of SENP3 phosphorylation functions as a switch for Cdc14b-APC/C^Cdh1^-Plk1 axis in controlling G2 checkpoint.

It is well known for protein SUMOylation involves DNA damage response^25,33^. However, how SENPs-mediated de-SUMOylation engages in DNA damage response is largely unknown. In our previous study, we showed that Cdk1 phosphorylates and inactivate SENP3-mediated de-SUMOylation at G2/M phase, which is essential for maintaining chromosomal stability in mitosis^32^. However, under genotoxic stress, p53 inhibits Cdk1 activation followed by inhibiting SENP3 phosphorylation. Importantly, p53-mediated the inhibition of SENP3 phosphorylation is essential for p53-induced G2 checkpoint responding to DNA damage. We show that expression of SENP3 phosphorylation mutant (SENP3 9A) alone can mimic p53-mediated DNA damage signaling to induce G2 checkpoint. We also show that the deficiency of SENP3 phosphorylation can promote APC/C^Cdh1^ ubiquitination activity through Cdc14b-mediated de-phosphorylation Cdh1. These data suggest that p53-mediated the inhibition of SENP3 phosphorylation would further promote p53 inhibition on Cdk1 via a positive feedback loop.

During cell cycle progression through G2/M phase, multiple proteins are SUMOylated^22,34^. In unperturbed cells, ubiquitin ligase APC/C^Cdh1^ is kept silence at G2 and early mitosis by Cdk1-mediated phosphorylation, but is activated after inactivation of Cdk1 and dephosphorylation at onset of anaphase^14^. However, when cells encounter DNA damage at the G2 phase, APC/C^Cdh1^ is activated to induce degradation of Plk1 and thereafter G2 arrest^16,17^. So far, three mechanisms have been proposed to explain reactivation of APC/C^Cdh1^ at the G2 phase upon DNA damage: (1) down-regulation of Cdk1 activity in response to DNA damage^17^; (2) p53-dependent long-term response to DNA damage by inactivation of APC/C inhibitor Emi1;^35^ (3) the translocation of Cdc14B phosphatase from the nucleolus to the nucleoplasm to dephosphorylate Cdh1^16^. Here we show that SENP3-mediated Cdh1 de-SUMOylation also engage in p53-induced DNA damage response. Yeast SUMOylation mutants showed a large budded phenotype and fail to properly degrade APC/C substrates Pds1 and Clb3, indicating an essential role for SUMOylation during the mitotic transition^36^. Recently, APC4, one subunit of APC/C complex, has been identified to be SUMOylated during mitosis and is required for timely anaphase onset^37,38^. Proteomic studies have also identified SUMOylation sites on other subunits of the APC/C^39^. These data suggest that SUMOylation could regulate APC/C activity.

We further identified Cdh1 as one target of SENP3 for de-SUMOylation in response to DNA damage at G2. SENP3-mediated de-SUMOylation of Cdh1 may create a favorable interface for binding to phosphatase Cdc14B, so that Cdc14B can efficiently remove inhibitory phosphate from Cdh1, which then reactivated for ubiquitination and degradation of Plk1 by proteasome and followed by G2 arrest. In this case, SUMOylation may act as an inhibitory signal that prevented interaction between phosphorylated Cdh1 and Cdc14B during unperturbed G2 and early mitosis to keep APC/C^Cdh1^ in an inactive state. Thus, SUMOylation and phosphorylation act as a double-lock mechanism in regulation of Cdh1 activation, and sequential SENP3-mediated de-SUMOylation and Cdc14b-mediated dephosphorylation of Cdh1, allowed efficient signal transduction of Cdh1-Plk1 axis to respond to G2 DNA damage.

## Materials and methods

### Reagents

Nocodazole (Cat#487928) and Thymidine (Cat#89270) were purchased from Sigma-Aldrich LLC. Doxorubicin hydrochloride (Cat#2252) was obtained from R&D Systems, Inc. Irinotecan (Cat#S2217), oxaliplatin (Cat#S1224), and p53 activator nutlin3a (Cat#S8059) were all from Selleck Chemicals. Monoclonal antibody to PLK1 was purchased from Thermo Fisher Scientific Company. Antibody against CDC14B (Cat#sc-374572), cdc2 p34 (Cat#sc-54) and chk1 (Cat#sc-8408) were from Santa Cruz Biotechnology. Anti-p53 (Cat#2524), anti-p21 (12D1) (Cat#2947), anti-phospho-cdc2 (Tyr15) (Cat#9111), anti-phospho-cdc2 (Thr161) (Cat#9114), anti-Claspin (Cat#2800), anti-p-chk1 (ser-317) (Cat#8191), and anti-p-histone H3 (ser-10) (Cat#9701) antibodies were all from Cell Signaling Technology. CDC25A (Cat#ab989) and CDH1 (Cat#ab217038) were purchased from Abcam. Dulbecco’s Modified Eagle Medium (DMEM), fetal bovine serum (FBS), Trypsin (0.25%), and Penicillin- Streptomycin- Glutamine (100×) were obtained from Thermo Fisher Scientific company.

### Cell culture and stable cell line construction

HCT116 p53 WT and p53 KO (Colon cancer) cell lines were purchased from American Type Culture Collection (Manassas, VA, USA). Cells were cultured in Dulbecco’s modified Eagle’s medium (DMEM; Gibco) plus 10% fetal bovine serum (FBS; Gibco) supplemented with 1% penicillin-streptomycin (Gibco) at 37 °C and 5% CO2. To generate stable transfection cell lines, SENP3 (WT) and SENP3 (9A) was cloned into the lentivector pCDH (System Biosciences, #CD510B-1). Production of Lentivirus and infection were carried out according to user manual as previously reported^32^. The cells were selected with 2 μg/mL puromycin (Amresco) for 1 week to get SENP3 (WT) and SENP3 (9A) stably transfected cells.

### Cell synchronization and drug treatment

For G1/S block, cells were treated with thymidine to a final concentration of 2 mM according to the method described previously^40^. For G2/M block, cultured cells were added with nocodazole to a final concentration of 100 ng/mL for 12 h after 3 h release from G1/S block. For drug treatment, cells were synchronized as described previously^16^. Pulse treatment of cells with doxorubicin at a final concentration of 0.5 mM was performed for 1 h.

### Western blotting and immunoprecipitation

The whole-cell extracts were prepared in RIPA buffer (20 mM Tris, 2.5 mM EDTA, 1% Triton X-100, 1% deoxycholate, 0.1% SDS, 40 mM NaF, 10 mM Na_4_P_2_O_7_, and 1 mM phenylmethylsulphonyl fluoride) supplemented with proteinase inhibitor cocktail (Calbiochem, San Diego, CA, USA). Lysates were then centrifuged at 12,000 rpm for 15 min to remove insoluble material. Supernatant was used for routine immunoprecipitation and western blotting. In total, 45 μg proteins from each treatment were resolved on SDS-polyacrylamide gels. Protein concentrations were determined using bicinchoninic acid assay and equalized before loading. After electrophoresis, the proteins were electrotransferred onto a PVDF membrane, blocked with 5% Bovine Serum Albumin (BSA) and probed with primary antibodies overnight at 4 °C, then exposure to horseradish peroxidase-conjugated secondary antibodies for 1 h and examined by chemiluminescence.

### Cell-cycle analysis

FACS analysis for cell cycle was performed using propidium iodide DNA staining (Biolegend) by following manufacturer’s protocol. To analyze mitotic cell cycle entry, cells were fixed and stained with propidium iodide and an antibody against phospho-Histone H3 (Ser10), followed by FITC-conjugated secondary antibody (Molecular Probes). The cellular DNA content and the percentages of M phase cells were determined by flow cytometry using a FACS Calibur flow-cytometer (BD Biosciences) and analyzed by Flow Jo software (Tree Star Inc., Stanford) as described^41^.

### Statistical analysis

All the experiments have been carried out in triplicate and repeated twice. All data presented were analyzed with one-way ANOVA, followed by Student’s t-test. *P*-values ≤ 0.05 were considered statistically significant.

## Supplementary information

supplementary information

## Supplementary information

**Supplementary Information** accompanies the paper at (10.1038/s41421-020-0154-2).
