# Supplementary material for: P53 suppresses SENP3 phosphorylation to mediate G2 checkpoint
Source: Cell Discov. 2020 Apr 21;6:21. doi: 10.1038/s41421-020-0154-2 (PMC7171148; doi:10.1038/s41421-020-0154-2)

Supplementary Information

Supplementary Figure Legends

Supplementary Fig. S1. P53-mediated suppression of SENP3 phosphorylation at G2/M phase upon DNA damage. (**a**) SENP3, but not SENP5, was highly phosphorylated at the G2/M phase. (**b**) HCT116 p53 WT cells were transfected with p53 siRNA for 48 hours, and then cells were treated as in Fig. 1a. Whole cell extracts were analyzed by western blot with antibodies to the indicated proteins.

Supplementary Fig. S2. Cdh1 is modified by SUMO1 at K96. (**a**) 293T cells were transfected with the indicated plasmids. After 48 hours cells were lysed and immunoprecipitation was performed with Flag antibody and western blot was performed with HA or Flag antibody. (**b**) HCT116 p53 WT/KO cells were lysed and immunoprecipitation was performed with Cdh1 antibody and western blot was performed with SUMO1 antibody. (**c**) 293T cells were transfected with the indicated plasmids. After 48 hours cells were lysed and immunoprecipitation was performed with Flag antibody and western blot was performed with HA or Flag antibody.

Supplementary Fig. S3. Interaction between Cdh1 and Cdc14b is reduced by Cdh1 SUMOylation. (**a**) SUMOylation of APC4/7 is not affected by doxorubicin. (**b**) 293T cells were transfected with the indicated plasmids and were treated as in Fig. 4d. Cells were lysed and immunoprecipitation was performed with HA antibody and western blot was performed with Flag antibody. (**c**) HCT116 p53 WT cells infected with retroviruses encoding either SENP3 9E or SENP3 WT, or SENP3 9A were further transfected with Flag-Cdc14B and HA-Cdh1 plasmids. Cells were synchronized at G2 phase and then subjected to immunoprecipitation and immunoblot analysis with antibodies to the indicated proteins.

Supplementary Fig. S4. Loss of SUMOylation activates APC/CCdh1. HCT116 p53 KO cells infected with retroviruses encoding indicated plasmids were treated as in Fig. 4e. Whole cell extracts were analyzed by western blot with antibodies to the indicated proteins.

Supplementary Fig. S1

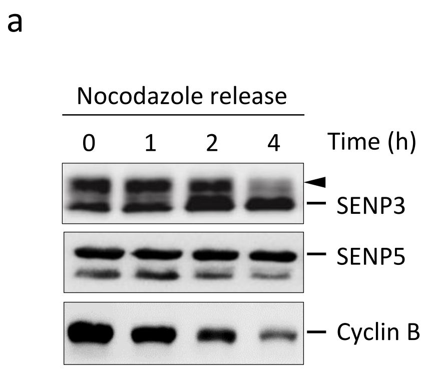

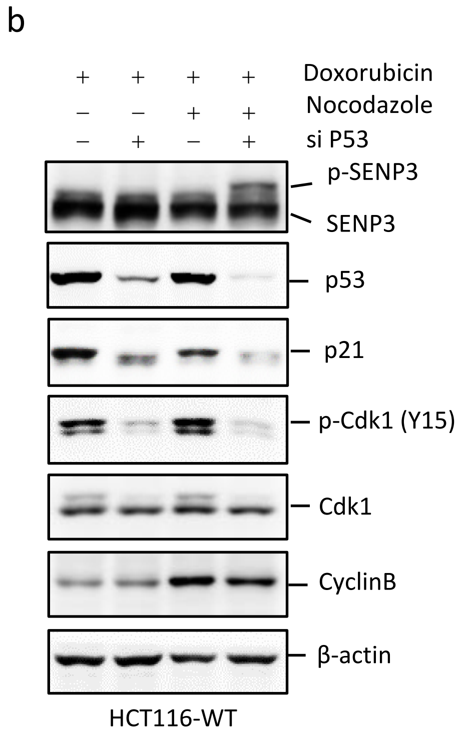

Supplementary Fig. S2

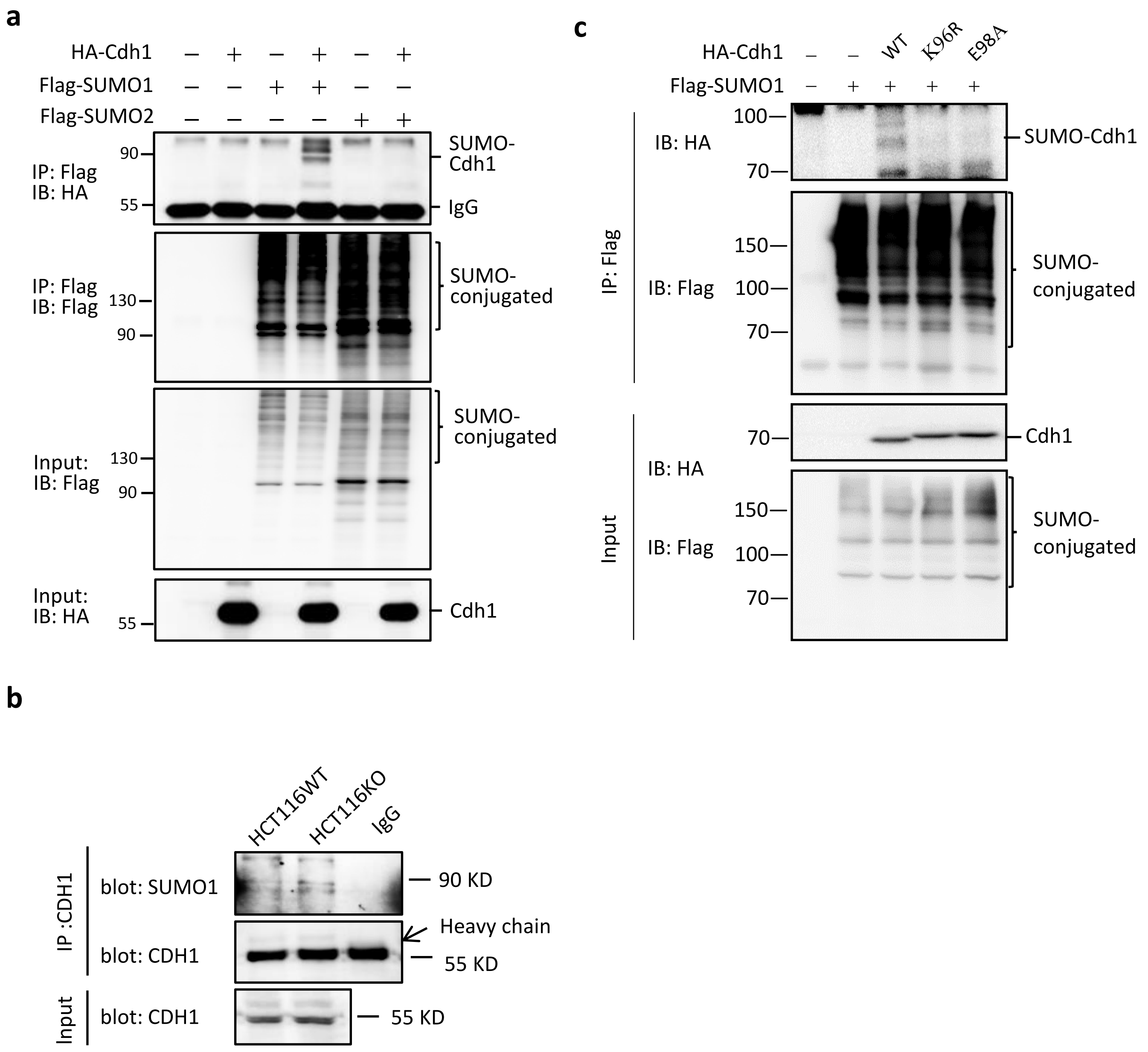

Supplementary Fig. S3

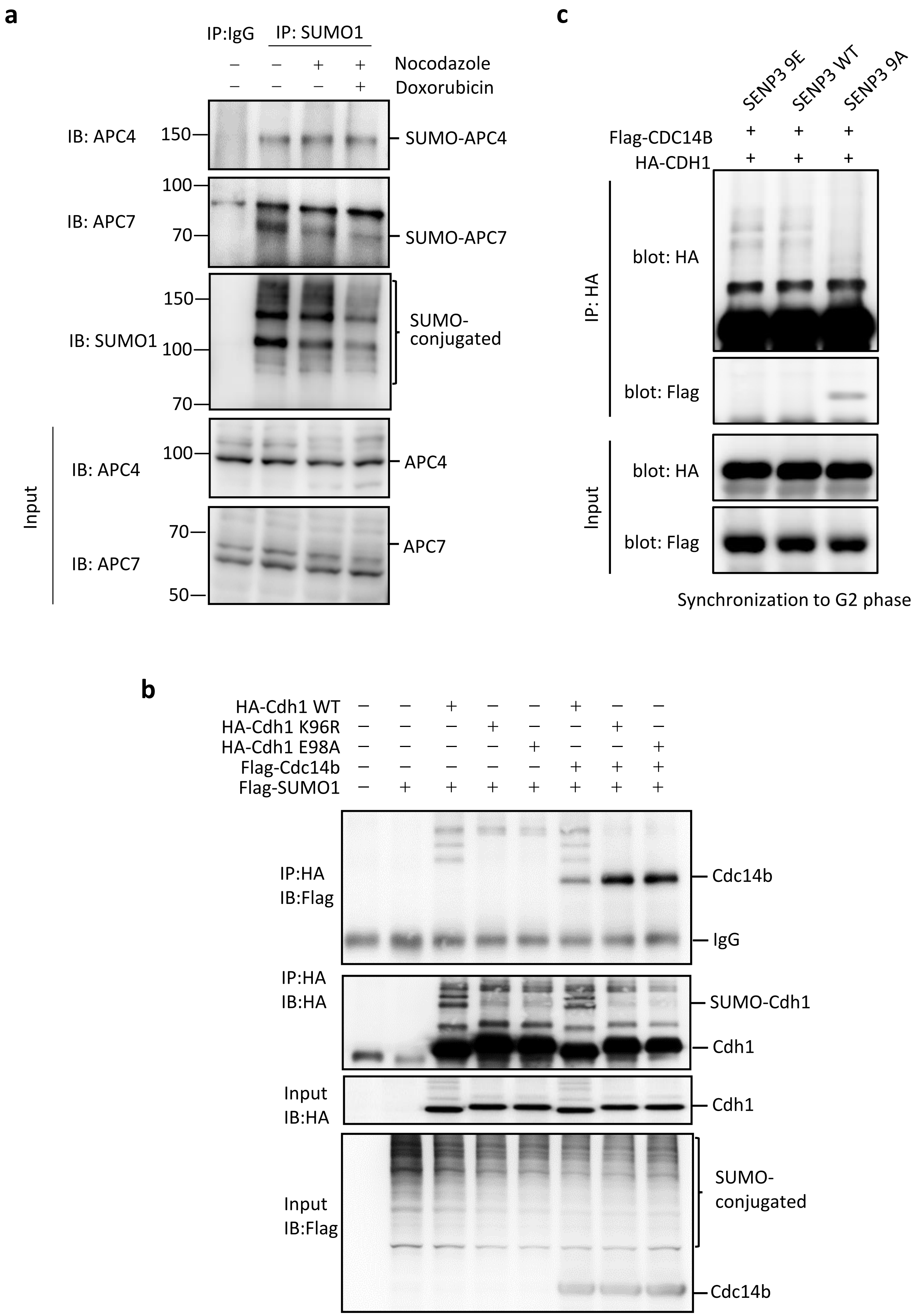

Supplementary Fig. S4

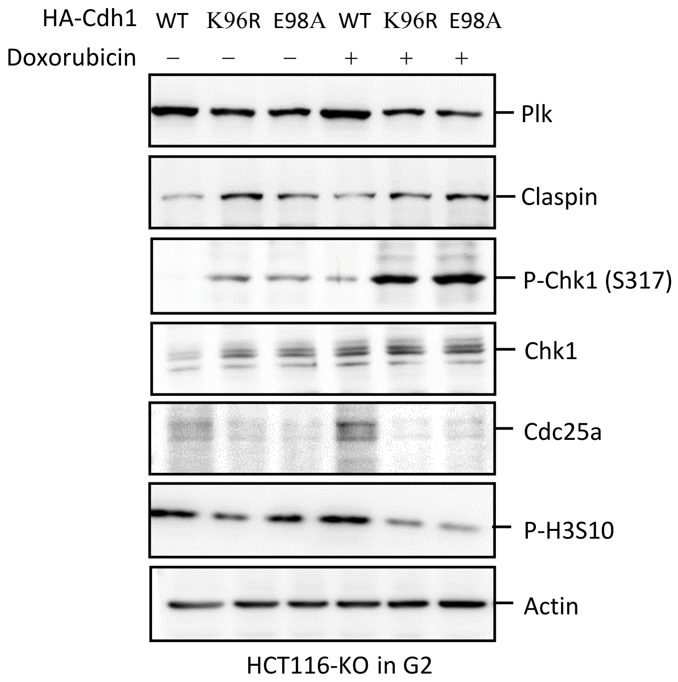

Supplement: Supplementary file 1 — supplementary information [file 41421_2020_154_MOESM1_ESM.pdf]
